# Draft Genome Sequence of *Flavobacterium* sp. Strain TAB 87, Able To Inhibit the Growth of Cystic Fibrosis Bacterial Pathogens Belonging to the *Burkholderia cepacia* Complex

**DOI:** 10.1128/genomeA.00410-16

**Published:** 2016-05-19

**Authors:** Luana Presta, Ilaria Inzucchi, Emanuele Bosi, Marco Fondi, Elena Perrin, Elisangela Miceli, Maria Luisa Tutino, Angelina Lo Giudice, Donatella de Pascale, Renato Fani

**Affiliations:** a Department of Biology, University of Florence, Florence, Italy; b Institute of Protein Biochemistry, National Research Council, Naples, Italy; c Department of Chemical Sciences, University of Naples Federico II, Naples, Italy; d Institute for the Coastal Marine Environment, National Research Council (IAMC-CNR), Messina, Italy; e Department of Biological and Environmental Sciences, University of Messina, Messina, Italy

## Abstract

We report here the draft genome sequence of the *Flavobacterium* sp. TAB 87 strain, isolated from Antarctic seawater during a summer campaign near the French Antarctic station Dumont d’Urville (60°40′S, 40°01′E). It will allow for comparative genomics and the fulfillment of both fundamental and application-oriented investigations. It allowed the recognition of genes associated with the production of bioactive compounds and antibiotic resistance.

## GENOME ANNOUNCEMENT

Antarctic bacteria are cold-adapted microorganisms that have evolved peculiar features to overcome barriers for growth at low temperatures. They are driving scientific interest, both in the field of ecological sciences as they play a key role in maintaining proper ecosystem functions, and in the clinical setting, since they are known to produce molecules able to exert antibacterial activity in order to withstand strongly adapted competitors. Indeed, it was recently demonstrated that many Antarctic bacteria exhibited the ability to counteract the growth of other Antarctic strains (1) and, more intriguingly, of some human pathogens belonging to the *Burkholderia cepacia* complex (BCC) (2–5), which represent a serious threat among immunocompromised patients, especially those affected by cystic fibrosis (CF).

Here, we report the draft genome sequence of *Flavobacterium* sp. strain TAB 87, a Gram-negative bacterium belonging to the family *Flavobacteriaceae* (6). The strain was isolated from seawater during a summer campaign near the French Antarctic station Dumont d’Urville (60°40′ S, 40°01′ E). The genome analysis of this Antarctic strain enables both fundamental and application-oriented investigations. Indeed, this strain completely inhibited the growth of 40 BCC strains belonging to 18 different bacterial species, most of which belonged to the species *Burkholderia cenocepacia* and *Burkholderia multivorans*, two of the most important CF pathogens. Moreover, some of the antimicrobial compounds produced were volatile organic compounds (VOCs), according to previous observations (3, 4). The draft genome sequence of *Flavobacterium* sp. TAB 87 was determined by the Institute of Applied Genomics and IGA Technology Services Srl (University of Udine, Italy) through a paired-end approach using an Illumina (Solexa) Genome Analyzer II platform. A total of 19,040,534 paired-end reads (average coverage, 1,004×) were initially obtained, those with low quality were trimmed with Streaming Trim (7), and those remaining were assembled with SPAdes genome assembler version 3.6.1 (8), which generated a total of 5,056 contigs. Those contigs <1,000 bp were discarded, while the others were embedded in the final version of the draft genome, which is 3,827,405 bp long and harbors 38 contigs (the longest of which is 1,014,695 bp long). The G+C content is 65.5%, similar to that of other *Flavobacterium* genomes sequenced so far. Annotation was performed by using Prokka (9), which, among all the predicted genes (3,365), identified a total of 3,323 protein-coding genes, 3 rRNA-coding genes, and 39 tRNA-coding genes.

Moreover, we screened the genome sequence for the presence of genetic traits involved in secondary metabolite biosynthesis. The analysis was performed within antiSMASH shell (10), revealing that the *Flavobacterium* sp. TAB 87 genome harbors four interesting gene clusters: a type I and a type III polyketide synthase (PKS) and two terpene biosynthetic gene clusters. Additionally, the genome sequence was analyzed through CARD (11), leading to the identification of *cfrA* and *Staphylococcus aureus* parE, two genes conferring resistance to florfenicol and fluoroquinolones, respectively.

### Nucleotide sequence accession numbers.

This whole-genome shotgun project has been deposited at GenBank under the accession no. LLWK00000000. The version described in this paper is version LLWK01000000.
